# Supplementary material for: Surface coupling in Bi2Se3 ultrathin films by screened Coulomb interaction
Source: Nat Commun. 2023 Jul 21;14:4424. doi: 10.1038/s41467-023-40035-0 (PMC10362050; doi:10.1038/s41467-023-40035-0)
Supplement: Supplementary file 1 — Supplementary information [file 41467_2023_40035_MOESM1_ESM.pdf]

Supplementary Information for

Surface coupling in Bi_2Se_3 ultrathin films by screened Coulomb interaction

Jia-nan Liu[1,2], Xu Yang[1,3], Haopu Xue[1,2], Xue-song Gai[1], Rui Sun[1,2], Yang Li[1,2], Zi-Zhao Gong[1,2], Na Li[1,2], Zong-Kai Xie[1,2], Wei He[1], Xiang-Qun Zhang[1], Desheng Xue[4] and Zhao-Hua Cheng[1,2,3,*]

1. *State Key Laboratory of Magnetism and Beijing National Laboratory for Condensed Matter Physics, Institute of Physics, Chinese Academy of Sciences, Beijing 100190, China*
2. *School of Physical Sciences, University of Chinese Academy of Sciences, Beijing 100049, China*
3. *Songshan Lake Materials Laboratory, Dongguan, Guangdong 523808, China*
4. *Key Laboratory for Magnetism and Magnetic Materials of the Ministry of Education, Lanzhou University, 730000 Lanzhou, China*

Content

Supplementary Note 1. The Form of Interaction

Supplementary Note 2. Self-consistent Gap Equation

Supplementary Note 3. Fitting Procedure

Supplementary Note 1. The Form of Interaction

The distance between two electrons in the top and bottom surface of Bi_2Se_3 ultrathin film with thickness of d is $\sqrt{d^2+|\mathbf{r}-\mathbf{r}'|^2}$, where $\mathbf{r}$ **and** $\mathbf{r}'$ are the position vectors projected on the surface. Therefore, the Coulomb interaction between these two electrons is Eq. (3) in the main text. However, it is hard to find the exact expression for the Fourier transform of V about $(\mathbf{r}-\mathbf{r}')$. We find an approximate expression

$$\mathcal{F}\left[\frac{1}{\sqrt{r^2+d^2}}e^{-\lambda^{-1}\sqrt{r^2+d^2}}\right] \approx \frac{1}{\sqrt{q^2+\lambda^{-2}}}e^{-d\sqrt{q^2+\lambda^{-2}}}. \qquad \text{(Supplementary Equation 1)}$$

The difference between this approximate result and the numerical result is shown in Supplementary Figure 1, and the relative error is less than 10^{-5}. Then, we get

$$V(d,q) = g^2\frac{1}{\sqrt{q^2+\lambda^{-2}}}e^{-d\sqrt{q^2+\lambda^{-2}}}, \qquad \text{(Supplementary Equation 2)}$$

where $g^2 = e^2/4\pi\varepsilon_0 \approx 14.4\ \mathrm{eV}\cdot\text{Å}$. For any function $f(q)$ converges when $q \to +\infty$, and after introducing the Lorentzian peak function with FWHM 2γ, $P_L(x,\gamma) \equiv \frac{1}{\pi}\frac{\gamma}{x^2+\gamma^2}$, one can find that

$$\begin{aligned}\int \frac{\mathrm{d}^2\mathbf{q}}{(2\pi)^2}V^2(d,q)f(q) &= g^4\int_0^{+\infty}\frac{\mathrm{d}q}{2\pi}\frac{q}{q^2+\lambda^{-2}}e^{-2d\sqrt{q^2+\lambda^{-2}}}f(q)\\ &= -\frac{g^4}{4}\int_0^{+\infty}\mathrm{d}q\frac{q^2+\lambda^{-2}}{\lambda^{-1}}\left[\frac{\partial}{\partial q}P_L(q,\lambda^{-1})\right]e^{-2d\sqrt{q^2+\lambda^{-2}}}f(q)\\ &= \frac{g^4}{4\pi}e^{-2\lambda^{-1}d}f(q=0)+\frac{g^4}{4\lambda^{-1}}\int_0^{+\infty}\mathrm{d}qP_L(q,\lambda^{-1})\partial_q\left[(q^2+\lambda^{-2})e^{-2d\sqrt{q^2+\lambda^{-2}}}f(q)\right],\end{aligned}$$

where partial integration is used in the last line. In our case, $\lambda^{-1} \approx 0.03\ \text{Å}^{-1}$, which makes the Lorentzian peak approximately become a delta function, and

$$\int \frac{\mathrm{d}^2\mathbf{q}}{(2\pi)^2}V^2(d,q)f(q) \approx \frac{g^4}{4}\left[\frac{1}{\pi}f(q=0)+\lambda^{-1}\frac{\partial}{\partial q}|_{q=0}f(q)\right]e^{-2\lambda^{-1}d}. \qquad \text{(Supplementary Equation 3)}$$

The exponential decay in Supplementary Equation 2 makes the error introduced by this approximation mainly originates from the fluctuation of $f(q)$ in the range of $0 < q < \lambda^{-1}$. This error will be further discussed in the next section.

Supplementary Note 2. Self-consistent Gap Equation

The free propagator of the Lagrangian described by Eq. (1) in the main text is

$$S_0(ik_n, \mathbf{k}) = (ik_n\gamma_0 - \hbar v_F \boldsymbol{\gamma} \cdot \mathbf{k})^{-1}, \qquad \text{(Supplementary Equation 4)}$$

where the Matsubara frequency $ik_n = i(2n+1)\pi/\beta,\ \beta = 1/k_B T$ is used. After adding the interaction term described by Eq. (2) in the main text, the generating functional of the propagator is

$$Z[\eta_\alpha, \bar{\eta}_\alpha] = \int \prod_{\alpha=\pm} \mathcal{D}\psi_\alpha \mathcal{D}\bar{\psi}_\alpha \exp\left[\frac{i}{\hbar}\int \mathrm{d}^3x(\mathcal{L}_0 + \bar{\psi}_\alpha\eta_\alpha + \bar{\eta}_\alpha\psi_\alpha)\right] \exp\left[\frac{i}{\hbar}\int \mathrm{d}^3x\mathcal{L}_{\mathrm{int}}\right],$$

where $\mathcal{L}_{0,\mathrm{int}}$ is the Lagrangian density of $L_{0,\mathrm{int}} = \int \mathrm{d}^3x\, \mathcal{L}_{0,\mathrm{int}}$, η_α are sources. This Coulomb interaction and the non-zero Fermi energy modify the Supplementary Equation 4 to the complete propagator[1]

$$S(ik_n, \mathbf{k}) = [\gamma_0(ik_n - \mu_0)A_1(k) - \hbar v_F \boldsymbol{\gamma} \cdot \mathbf{k} A_2(k) - m(k)]^{-1},$$

where μ_0 is the Fermi energy. $m(k)$ is the dynamical fermion mass, and $A_{1,2}$ is denoted as the wave-function renormalization functions. One can get the 2-point propagator by $S_\alpha(x,y) \equiv S_{\alpha,\alpha}(x,y) = \left[\frac{\delta^2 Z}{\delta\bar{\eta}_\alpha(x)\delta\eta_\alpha(y)}\right]_{\eta=0}$. After transforming the Fourier from space-time to energy-momentum space, the first order of non-zero self-energy $\Sigma_\alpha(k_0, \mathbf{k}) = S_{0_\alpha}^{-1} - S_\alpha^{-1}$ is the Hartree-like term

$$\Sigma_{H_\alpha} = V(q=0, d)\rho_{-\alpha},$$

and the second order self-energy is

$$\Sigma_{2_\alpha}(ik_n, \mathbf{k}) = -\frac{2}{\beta}\sum_m \int \frac{\mathrm{d}^2\mathbf{q}}{(2\pi)^2} V^2(d,q)\gamma_0 S_\sigma(ik_n - iq_m, k-q)\gamma_0\, \Pi_{-\alpha}(iq_m, \mathbf{q}),$$

where $\Pi_\alpha(iq_m, \mathbf{q})$ is the polarization function as described by Eq. (7) in the main text. Since we only concern with the mass gap at Γ point, followed by the widely used approximation to solve interaction-induced mass gap problems in the Dirac system, we set $A_{1,2} = 1$ [1, 2, 3, 4], and treat $m(k)$ as a constant[3, 4, 5]. This treatment allows us to take the trace of m, and consequently obtain the self-consistent gap equation $m = \frac{1}{N_\gamma}\mathrm{Tr}\, \Sigma_{2_\alpha}(ik_n = 0, \mathbf{k} = 0)$, where N_γ is the rank of chosen gamma matrices.

The polarization function with $\mu_0 \neq 0$ of Dirac fermion has been studied in the problem of magnetic field-induced phase transition[2]. However, the static screening effect was concerned in their study and they treated

the static polarization function as a real number $\Pi \rightarrow \frac{1}{N_\gamma}\mathrm{Tr}\,\Pi(0,\boldsymbol{q})$, which may not be suitable for our case. Use the Feynman parameterization,

$$\Pi_\alpha(iq_m,\mathbf{q}) = -\int_0^1 \mathrm{d}x \int \frac{\mathrm{d}^2\mathbf{p}}{(2\pi)^2}\frac{1}{\beta}\sum_l \left\{\frac{1}{(ip_l-\mu_0)^2-\hbar^2 v_F^2\mathbf{p}^2-B^2} + \frac{2v_F^2\mathbf{p}^2+A+B^2}{[(ip_l-\mu_0)^2-\hbar^2 v_F^2\mathbf{p}^2-B^2]^2}\right\}, \qquad \text{(Supplementary Equation 5)}$$

where

$$\begin{cases} A = -x(1-x)q_m^2 - iq_m\mu_0(1-2x) - x(1-x)\hbar^2 v_F^2\mathbf{q}^2 + m^2 \\ \quad +m\hbar v_F\boldsymbol{\gamma}\cdot\mathbf{q} + m[(1-2x)iq_m - 2\mu_0]\gamma_0 + [2x(1-x)iq_m + (1-2x)\mu_0]\hbar v_F\boldsymbol{\gamma}\cdot\mathbf{q}\gamma_0\,. \\ B^2 = x(1-x)q_m^2 + x(1-x)\hbar^2 v_F^2\mathbf{q}^2 + m^2 \end{cases}$$

Use the identity $\sum_{n=0}^{\infty}\frac{1}{(n+X)^2+Y^2} = \frac{1}{2Y}[\psi(X+iY)-\psi(X-iY)]$ where ψ is the digamma function, the first term of Supplementary Equation 5 is

$$\begin{aligned} S_1 &\equiv \frac{1}{\beta}\sum_l \frac{1}{(ip_l-\mu_0)^2-\hbar^2 v_F^2\mathbf{p}^2-B^2} \\ &= -\frac{1}{i\pi Y}\left[\psi\left(\frac{1}{2}+i\frac{\mu_0+Y}{2\pi\beta^{-1}}\right)-\psi\left(\frac{1}{2}+i\frac{\mu_0-Y}{2\pi\beta^{-1}}\right)+(\mu_0\rightarrow-\mu_0)\right] \\ &= -\frac{1}{4}\frac{1}{Y}\left\{\tanh\left[\frac{1}{2}\beta(Y+\mu_0)\right]+(\mu_0\rightarrow-\mu_0)\right\} \end{aligned}$$

where $Y=\sqrt{\hbar^2 v_F^2\mathbf{q}^2+B^2}$. The second term of Supplementary Equation 5 is

$$\begin{aligned} S_2 &\equiv \frac{1}{\beta}\sum_l \frac{\hbar^2 v_F^2\mathbf{p}^2+A+Y^2}{[(ip_l-\mu_0)^2-Y^2]^2} \\ &= \frac{v_F^2\mathbf{p}^2+A+Y^2}{2Y}\frac{\partial}{\partial Y}S_1 \\ &= -\frac{v_F^2\mathbf{p}^2+A+Y^2}{8Y^2}\left\{\frac{\beta}{2}\mathrm{sech}^2\left[\frac{1}{2}\beta(Y+\mu_0)\right]-\frac{1}{Y}\tanh\left[\frac{1}{2}\beta(Y+\mu_0)\right]+(\mu_0\rightarrow-\mu_0)\right\}. \end{aligned}$$

Notice that when calculating the integral about $\mathbf{p}$ in Supplementary Equation 5, the only related term above is $\hbar^2 v_F^2\mathbf{p}^2$, which makes the integration not very complicated and the result is

$$\Pi_\alpha = \frac{1}{4\pi v_F^2}\int_0^1 \mathrm{d}x\left\{\frac{2}{\beta}\ln 2\cosh\left[\frac{1}{2}\beta(B+\mu_0)\right]-\frac{C}{2B}\tanh\frac{\beta}{2}(B+\mu_0)+(\mu_0\rightarrow-\mu_0)\right\},$$

where $C=A+B^2$. The correctness of the calculation above can be checked by comparing the $\frac{1}{N_\gamma}\mathrm{Tr}\,\Pi_\alpha(iq_m=0,\mathbf{q})$ and the results in previous work[2]. If define

$$\varpi_\beta(B) \equiv \frac{1}{\beta}\ln[2\cosh\beta B + 2\cosh\beta\mu_0],$$

it is easy to find that

$$\Pi_\alpha(iq_m, \mathbf{q}) = \frac{1}{8\pi\hbar^2 v_F^2}\int_0^1 \mathrm{d}x\left[2\varpi_\beta(B) - \frac{C}{B}\frac{\partial}{\partial B}\varpi_\beta(B)\right].$$

In our case, $\beta^{-1} \approx k_B \times 5\ \mathrm{K} \approx 0.43$ meV, which is much smaller than any energy scale we are studying. Therefore, the approximation of low temperature is reasonable, which makes

$$\varpi_\beta(B) \simeq \begin{cases} B & \mathrm{Re}\,B > \mu_0 \\ -B & \mathrm{Re}\,B < -\mu_0 \\ \mu_0 & -\mu_0 < \mathrm{Re}\,B < \mu_0 \end{cases},$$

and

$$\frac{\partial}{\partial B}\varpi_\beta(B) \simeq \begin{cases} 1 & \mathrm{Re}\,B > \mu_0 \\ -1 & \mathrm{Re}\,B < -\mu_0 \\ 0 & -\mu_0 < \mathrm{Re}\,B < \mu_0 \end{cases}.$$

Notice that B is always larger than zero, and in the region of $\mathrm{Re}\,B < \mu_0$, $\Pi_\alpha = \frac{\mu_0}{4\pi\hbar^2 v_F^2}$. As discussed above, this platform of Π_α makes the approximation of Supplementary Equation 3 holds. Notice that Supplementary Equation 3 requires that only $q \approx 0$ contributes the integral of $\mathbf{q}$, and $m \ll \mu_0$. Therefore, to get to the region of $\mathrm{Re}\,B > \mu_0$, i.e., $q_m^2 \gtrsim \frac{\mu_0^2}{x(1-x)} \geq 4\mu_0^2$, $|q_m|$ must be larger than $2\mu_0$, which is larger than the energy range of the whole Dirac cone, and this case is therefore ignored. Finally, the self-consistent gap equation becomes

$$\begin{aligned} m &\approx -\frac{\mu_0}{2\pi\hbar^2 v_F^2}\int \frac{\mathrm{d}^2\mathbf{q}\mathrm{d}q_0}{(2\pi)^3} V^2(q,d)\frac{m}{(-iq_0-\mu_0)^2 - (m^2 + \hbar^2 v_F^2 q^2)} \\ &\approx -\frac{\mu_0 g^4}{8\pi\hbar^2 v_F^2}e^{-2\lambda^{-1}d}\int \frac{\mathrm{d}q_0}{2\pi}\frac{m}{(-iq_0-\mu_0)^2 - m^2} \\ &= \frac{1}{2}\frac{\mu_0 g^4}{8\pi\hbar^2 v_F^2}e^{-2\lambda^{-1}d}, \qquad \text{(Supplementary Equation 6)} \end{aligned}$$

where Supplementary Equation 3 has been used in the second approximately equal sign. The influence of the Hartree term can be also considered by the transformation $\mu_0 \to \mu = \mu_0 + \Sigma_H$, and $E_{\mathrm{gap}} = 2m$. Notice that the fluctuation of $f(q)$ near $q = 0$ in Supplementary Equation 3 comes from the Green function in the first line of Supplementary Equation 6, the error of delta-function approximation is less than $\frac{\hbar^2 v_F^2 \lambda^{-2}}{\mu_0^2} \approx 3.5\%$.

Supplementary Note 3. Fitting Procedure

The angle-resolved photoemission spectroscopy (ARPES) can detect the imaginary part of the Green function, i.e., the spectral function $A(\mathbf{k},\omega)$ [6]. In order to visually find the energy band, the curvature distribution of the ARPES spectra of 4-6 QL is calculated by the method of P. Zhang, *et al*[7], which are shown in Supplementary Figure 2a-c. The abrupt change of velocity near the Dirac point confirms the existence of the mass gap below 6 QL. By cutting $A(\mathbf{k},\omega)$ in the direction of k or ω, one can get the momentum distribution curves (MDCs) or the energy distribution curves (EDCs), respectively. Assuming the self-energy does not change a lot, one can precisely get the energy band $\omega(\mathbf{k})$ by fitting the MDCs or EDCs with Lorentzian peak [7,8,9]. The magnitude of energy gaps is further extracted by the EDCs at the Γ point as shown in Supplementary Figure 4. Since the gaps are small near 5 QL, besides the fitting error of the peak position, we also added the full width at half-maximum (FWHM) of the fitting results as the additional error in the error bars in Fig. 3. Since the velocity goes from zero to quite large when k moves away from the Dirac point, we fit the MDCs (as shown in Supplementary Figure 2d-j) and the EDCs (as shown in Supplementary Figure 2k-o) when the corresponding velocity is large and small, respectively. The fitting results are shown as the red short line in Supplementary Figure 2d-o, which are the black dots in Fig. 2 in the main text. The same method is used to analysis the ARPES spectra of 1.5 and 2.5 QL Bi_2Se_3 as shown in Supplementary Figure 3. As shown in Supplementary Figure 5, the AFM results of 2.5 QL shows the islands and steps with a height of 1 QL. This result accounts that the APRES results should be a superposition of the results between two integer layers.

Supplementary Figure 1

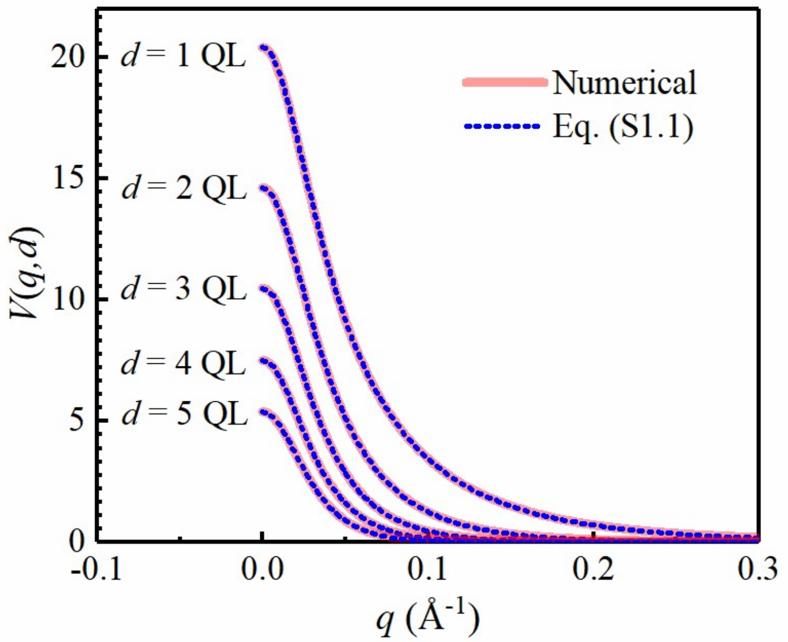

Supplementary Figure 1. The numerical verification of Supplementary Equation 1 with $\boldsymbol{\lambda = 28.5}$ Å and different $\boldsymbol{d}$. The thick light red lines are the numerical result of the Fourier transform described as the left side of Supplementary Equation 1, while the blue dot lines are the right side of Supplementary Equation 1. The relative error is less than 10^{-5}.

Supplementary Figure 2

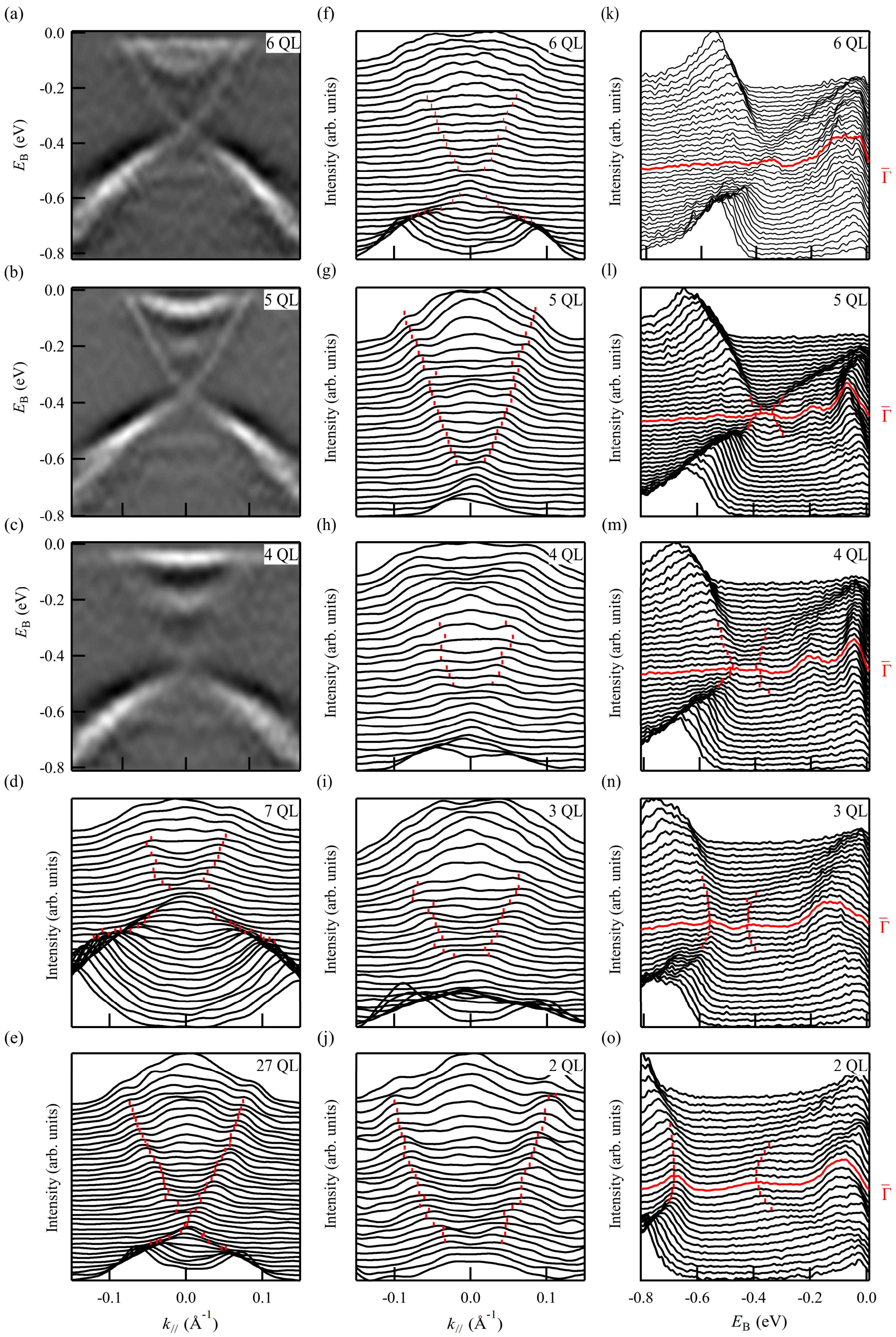

Supplementary Figure 2. The peak positions fitted from ARPES spectra of different thicknesses Bi_2Se_3. **a-c** are the improved ARPES spectra of 4-6 QL Bi_2Se_3 by the method of curvature distribution[7]. **d-j** and **k-o** are MDCs and EDCs of smoothed ARPES spectra of Bi_2Se_3 whose thickness is labeled at the right-top of each subfigure. The red short lines are the peak positions fitted by Lorentzian peak.

Supplementary Figure 3

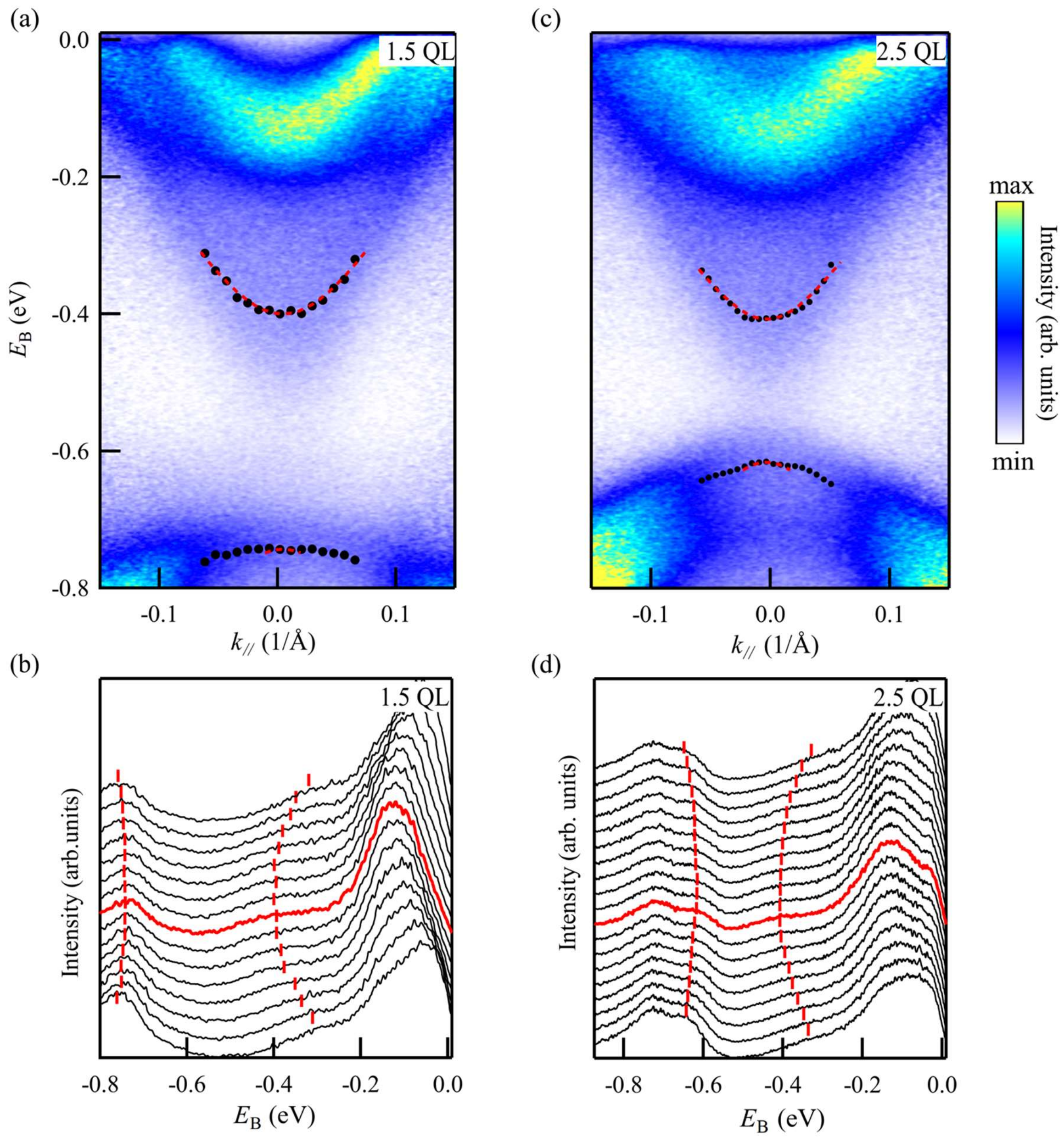

Supplementary Figure 3. The ARPES spectra of Bi_2Se_3 with the thickness of half-integer thicknesses. a and **c** are the ARPES spectra of 1.5 and 2.5 QL Bi_2Se_3 with their EDCs and Lorentzian fitting results shown in **b** and **d**, respectively.

Supplementary Figure 4

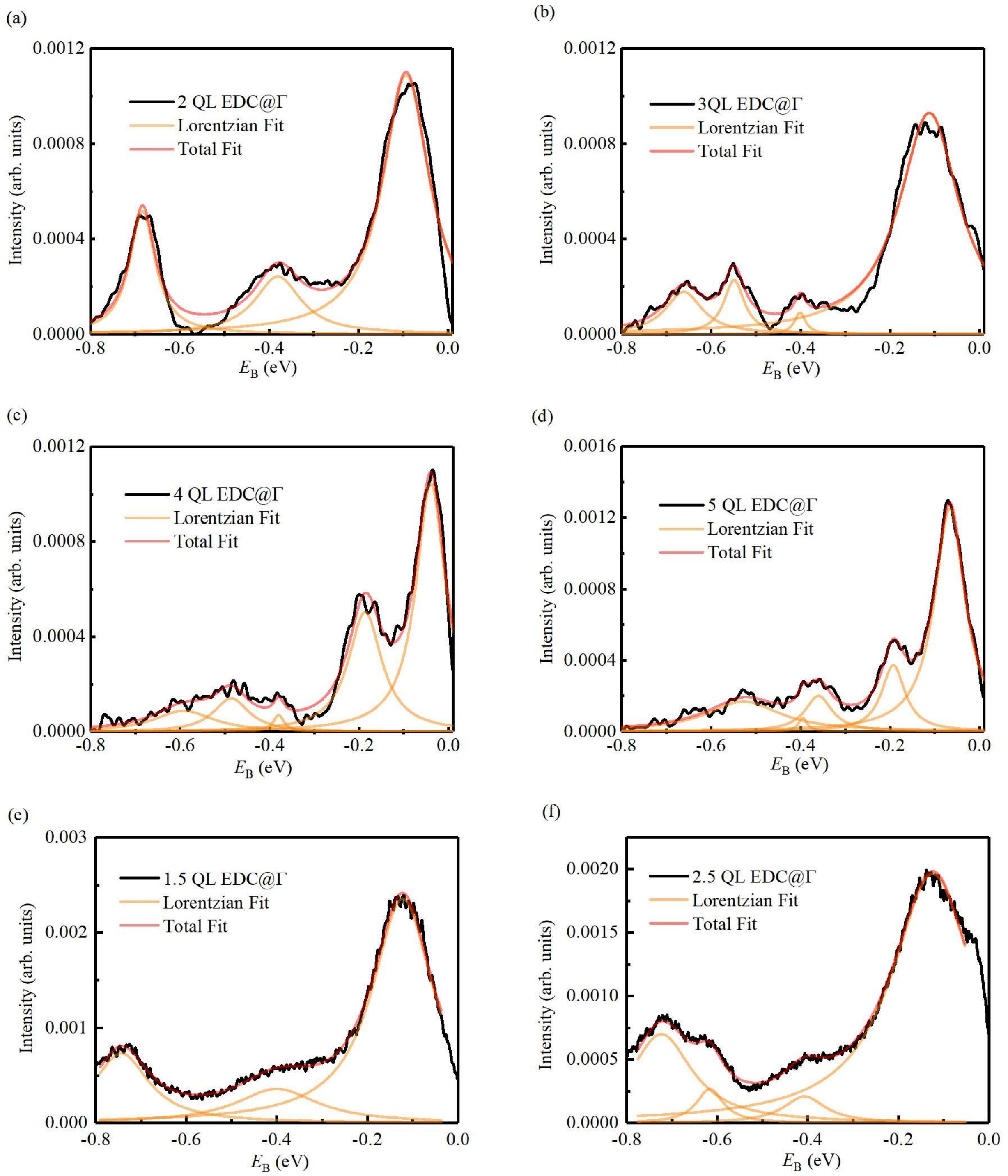

Supplementary Figure 4. The fitting results of EDCs at Γ point with different thicknesses. The black lines are the EDCs of the ARPES spectra, while the orange lines and red lines are the Lorentzian fitting of the single peak and the total EDCs. The FWHM of fitting results is also considered by adding a corresponding an additional error in the error bar in Fig. 3.

Supplementary Figure 5

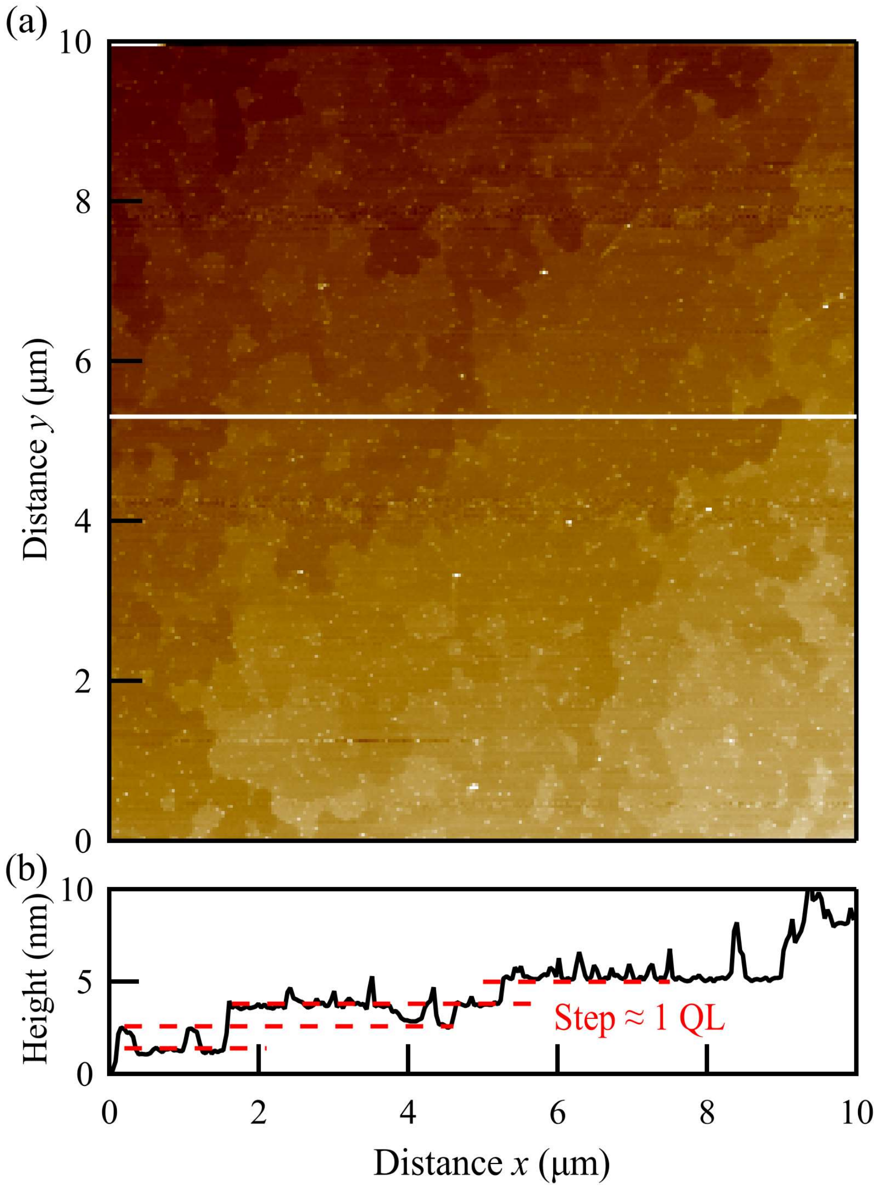

Supplementary Figure 4. (a) The AFM image of the Bi_2Se_3 film with the thickness of 2.5 QL. The scan size is $10 \times 10\ \mu m^2$, and the oblique background is removed to show the steps. (b) The cross profile along the write line in (a). The red dot lines in (b) are guide for the eye.

Supplementary References

1. Liu, G.-Z., Li, W. & Cheng, G. Interaction and excitonic insulating transition in graphene. *Phys Rev B* **79**, 205429 (2009).

2. Gorbar, E. V., Gusynin, V. P., Miransky, V. A. & Shovkovy, I. A. Magnetic field driven metal-insulator phase transition in planar systems. *Phys Rev B* **66**, 045108 (2002).

3. Appelquist, T. W., Bowick, M., Karabali, D. & Wijewardhana, L. C. Spontaneous chiral-symmetry breaking in three-dimensional QED. *Phys Rev D* **33**, 12 (1986).

4. Li, W. & Liu, G.-Z. Coulomb interaction and semimetal–insulator transition in graphene. *Phys Lett A* **374**, 2957 (2010).

5. Pisarski, R. D. Chiral-symmetry breaking in three-dimensional electrodynamics. *Phys Rev D* **29**, 10 (1984).

6. Damascelli, A. Probing the electronic structure of complex systems by ARPES. *Phys Scripta* **T109**, 61-74 (2004).

7. Zhang, P., *et al.* A precise method for visualizing dispersive features in image plots. *Rev Sci Instrum* **82**, 043712 (2011).

8. Valla, T., Fedorov, A. V., Johnson, P. D. & Hulbert, S. L. Many-body effects in angle-resolved photoemission: Quasiparticle energy and lifetime of a Mo(110) surface state. *Phys Rev Lett* **83**, 2085-2088 (1999).

9. Chen, Y. L., *et al.* Massive Dirac fermion on the surface of a magnetically doped topological insulator. *Science* **329**, 659-662 (2010).
